# Googling adenomyosis: a systematic review of internet-based information

**DOI:** 10.1590/1806-9282.20251982

**Published:** 2026-06-15

**Authors:** Beatriz Piaulino de Araújo, Clarissa Suzart Lopes da Silva, Cristina Laguna Benetti-Pinto, Daniela Angerame Yela

**Affiliations:** 1 Universidade de Campinas, School of Medical Sciences, Department of Obstetrics and Gynecology – Campinas (SP), Brazil.

## INTRODUCTION

Adenomyosis is characterized by the presence of endometrial tissue in the myometrium, associated with hypertrophy and hyperplasia in the adjacent myometrial tissue^1^. With an estimated prevalence of 20–34%, adenomyosis is the most common uterine disorder in women of reproductive age worldwide and is known to be a contributing factor to dysmenorrhea, pelvic pain, heavy menstrual bleeding, and subfertility^2^.

Globalization and the advent of the internet have granted widespread access to a vast array of content and information. This content encompasses multiple areas of knowledge, including health-related topics. Today, the internet has become a significant source of research for a large number of women seeking immediate clarification regarding their medical conditions^3,4^.

However, a crucial question arises: is this information truly reliable? It is essential that health consumers have access to high-quality, evidence-based information. In the healthcare context, lay texts discussing symptoms, treatments, and prognoses may inadvertently cause emotional distress and negatively affect the doctor–patient relationship^3^. The lack of clarity in such texts may foster distrust among women, leading them to question the medical diagnosis, proposed management, and treatment options^4,5,6,7^.

Poorly written lay texts that present adenomyosis treatments in a generalized and confusing manner can induce insecurity and anxiety in patients. Furthermore, women may be induced to initiate self-treatment without medical guidance, resulting in ineffective care, false expectations, and unnecessary financial burden^4,5,6,7^.

Given this context, the present study aims to evaluate the quality and accuracy of online medical information regarding the characteristics of adenomyosis.

## METHODS

A protocol was registered in the International Prospective Register of Systematic Reviews, number: CRD42023395332. This review was conducted in accordance with the Preferred Reporting Items for Systematic Reviews and Meta-Analyses statement^8^.

### Selection of publications

A systematic review was conducted of publications related to the term “Adenomyosis,” in both English and Portuguese, on World Wide Web websites during the period from January 1, 2023, to March 31, 2023, using a lay literature search platform (Google).

### Exclusion criteria

The following were excluded from analysis: duplicate texts, scientific or medical literature, advertisements, content from social media, unavailable or paid-access pages, exam-related questions, news articles, texts in other languages, repeated entries, and personal narratives, such as case reports.

### Classification of publications

Two reviewers (BPA and CLSA) were trained in the use of quality assessment tools. Accuracy was assessed based on a prioritized list of recommendations included in the Brazilian Federation of Gynecology and Obstetrics guidelines. All recommendations were extracted by two authors independently. Discrepancies were resolved by discussion. The lay literature texts were evaluated based on six key domains related to adenomyosis: definition, risk factors, signs and symptoms, diagnosis, clinical treatment, and surgical treatment. These domains were cross-referenced with current clinical guidelines (Table 1). Notably, two reviewers independently reviewed each website, assessing the accuracy of the information. Each domain was scored as follows: 0 (absent or incorrect), 1 (incomplete), or 2 (complete). Accuracy assessment was anchored between 0 and 12. Discrepancies were resolved by discussion.

**Table 1:** Key domains in adenomyosis.

Definition	A gynecological condition characterized by the infiltration of endometrial tissue into the myometrium, leading to an increase in uterine volume. These infiltrations may present in a diffuse or nodular pattern, the latter forming what are known as adenomyomas.
Risk factors	Age between 40 and 50 years; Prolonged estrogen exposure: early menarche (≤10 years), short menstrual cycles (≤24 days); use of combined oral contraceptives; body mass index (BMI) ≥25; parity ≥2 deliveries; use of tamoxifen; and history of uterine surgeries (e.g., cesarean section, curettage).
Clinical presentation	Abnormal uterine bleeding, dysmenorrhea, chronic pelvic pain, dyspareunia, infertility. Some women may remain asymptomatic.
Diagnosis	Clinical signs: enlarged and softened uterus on physical examination. Gold standard: histopathological evaluation, typically performed via hysterectomy specimen or biopsy obtained through hysteroscopy or laparoscopy. Adenomyosis is confirmed when endometrial tissue is observed within the myometrium at a minimum depth of 1–4 mm from the endometrial-myometrial junction, or when at least 25–33% of the myometrial thickness is involved. Imaging findings: In 2D TVUS, heterogeneous myometrial echotexture is noted, with poorly defined foci that generate echogenic striations on ultrasound, myometrial cysts, globular uterus, asymmetry between the anterior and posterior walls, and poor definition of the myometrial-endometrial junction. In 3D TVUS, enlargement or hyperplasia of the junctional zone is observed, which is an important finding for the early diagnosis of adenomyosis. In MRI, the most common findings are an enlarged and asymmetric uterus, abnormal myometrial signal intensity, thickening of the junctional zone, and T1 hyperintense foci indicating hemorrhage, and T2 hypointense areas indicating junctional zone thickening.
Pharmacological treatment	Nonsteroidal anti-inflammatory drugs (NSAIDs), continuous use of combined oral contraceptives or progestin-only therapies, levonorgestrel-releasing intrauterine device (IUD), gonadotropin-releasing hormone (GnRH) agonists (short-term use, up to 6 months, due to hypoestrogenic side effects), aromatase inhibitors, and danazol.
Surgical treatment	Specific information on: Hysteroscopic endometrial ablation and resection. Uterine artery embolization (devascularization of adenomyotic areas of the myometrium leads to symptom improvement). Myometrial electrocoagulation via laparoscopy or hysteroscopy (promotes reduction of adenomyotic foci through necrosis. Its advantage is that it can be used both in localized and disseminated disease). Ultrasound-guided MRI (ExAblate). HTA—definitive treatment: hysterectomy is the first option for patients who have completed their reproductive cycle and present with severe symptoms. Uterine removal can be performed via vaginal, laparoscopic, or abdominal approach.

Notably, two reviewers independently assessed each website using validated instruments, including assessments of credibility assessed by the White instrument, with a score between 0 (poor) and 10 (excellent); quality assessed by the DISCERN instrument; and readability assessed by the Flesch-Kincaid instrument, with a score between 0 (poor) and 100 (excellent). Discrepancies were resolved by a third reviewer (DAY).

The credibility of the websites was assessed by two reviewers independently using the validated instrument. This instrument, developed for consumers of health information, provides a set of criteria that can be used to accurately and reliably assess the quality of health information on the internet. Credibility was assessed using 10-point criteria: (1) source; (2) context; (3) currency; (4) usefulness; (5) editorial review process; (6) hierarchy of evidence; (7) statement of original source; (8) disclaimer, which included ownership, sponsorship, funding, and advertising; (9) omissions; and (10) feedback^9^.

Following the comparison, the quality level of each text was assessed using the DISCERN tool, which provides a score from 1 to 5 across 16 questions that evaluate the quality of health information provided. Based on DISCERN’s guidelines^10,11^, publications were classified as: High (scores 4 and 5): indicates the publication is of “good” quality—a useful and appropriate source of information regarding treatment options; Moderate (score 3): indicates the publication is of “fair” quality—a generally useful source of information but with notable limitations; additional information or support would clearly be required; and Low (scores 1 and 2): indicates the publication is of “poor” quality—contains serious shortcomings and is neither useful nor appropriate as a source of treatment-related information; unlikely to provide any benefit and should not be used.

Websites’ readability was assessed using the Flesch-Kincaid Reading Ease Test^12^. The Flesch-Kincaid score is generated from the following equation: 206.835 and 1.015 (total words/total sentences) and 84.6 (total syllables/total words) (www.readabilityscore.com). The scores were anchored between 0 (complex language) and 100 (simple language) and could be categorized by reading age or educational status: 90–100 (5th grade); 80–90 (6th grade); 70–80 (7th grade); 60–70 (8th and 9th grade); 50–60 (10th, 11th, and 12th grade); 30–50 (college); or 0–30 (college graduate). Discrepancies were resolved through discussion among reviewers.

## RESULTS

**Figure 1:**
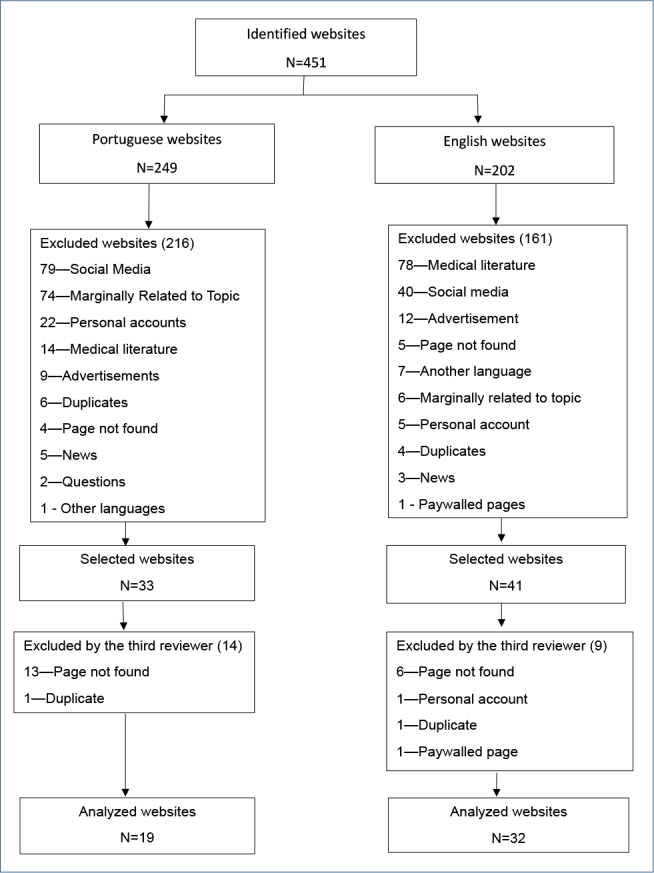
Flowchart of the selection of websites in the Portuguese and English language.

In the Portuguese language, 249 websites were analyzed, of which 216 were excluded by the initial reviewers. Of the remaining websites, 14 had disagreements and were excluded by a third reviewer. In total, 230 websites were excluded. The reasons for exclusion, in order of frequency, are listed below. In the English language, 202 websites were analyzed, of which 161 were excluded by the initial reviewers. Of the remaining websites, 9 had disagreements and were excluded by a third reviewer. In total, 170 websites were excluded. The reasons for exclusion, in order of frequency, are listed in Figure 1.

Of the 19 included websites, 12 sites (63.16%) received a score of 1 on DISCERN (low quality), 6 sites (31.58%) received a score of 3 (moderate quality), and only 1 site (5.26%) received a score of 5 (high quality). As for readability, all sites required a higher education level, with 12 sites requiring ongoing college and 7 sites requiring college graduates.

Only 6 out of 19 sites (31.6%) were considered accurate, while the other 13 were not accurate. Among the sites considered accurate (score of 8 or higher), 3 sites received a score of 10 (out of 12), 1 site scored 9, and 2 sites scored 8. Among the sites considered not accurate, 4 sites received a score of 7, 5 sites scored 6, 1 site scored 5, 1 site scored 4, and 2 sites scored 2. The average accuracy score of the sites in Portuguese was 6.631. Of the 19 sites, only 2 were considered credible, with 1 site scoring 8 (out of 10) and 1 site scoring 7. The remaining 17 sites were considered not credible: 3 sites scored 6, 2 sites scored 5, 9 sites scored 4, 2 sites scored 3, and 1 site scored 2. The average credibility score of the sites in Portuguese was 4.578. All Portuguese-language websites included a definition of adenomyosis and the symptoms. The risk factors were described in 16 of them. Diagnosis was mentioned on 16 websites. Regarding treatment, medical management was described on all websites, while surgical treatment was mentioned in 18.

While in Portuguese the main reason for exclusion was “social media,” in English it was “medical literature.” Of the 32 included websites, 18 sites (56.25%) received a score of 1 on DISCERN (low quality), 10 sites (31.25%) received a score of 3 (moderate quality), and 4 sites (12.5%) received a score of 5 (high quality) (Table 2). As for readability, all sites required a higher education level, with 10 sites requiring ongoing college and 22 sites requiring college graduates.

**Table 2:** Included websites in Portuguese and English language - DISCERN score.

Portuguese websites	Score	English websites	Score
https://www.procriar.com.br/blogprocriar/adenomiose-o-que-e-qual-tratamento/	1	https://www.uabmedicine.org/specialties/adenomyosis/#	1
https://educa.cetrus.com.br/adenomiose-o-diferencial-do-us-no-diagnostico/#:~:text=A%20 adenomiose%20%C3%A9%20uma%20 modifica%C3%A7%C3%A3o,endometriais%20 envoltos%20por%20hiperplasia%20e	1	https://www.shreeivfclinic.com/blogs/stages-of-adenomyosis/	1
https://drlucassantana.com/adenomiose/	1	https://sonacare.com.au/adenomyosis	1
https://www.msdmanuals.com/pt-br/profissional/ginecologia-e-obstetr%C3%ADcia/doen%C3%A7as-ginecol%C3%B3gicas-diversas/adenomiose-uterina	1	https://timesofindia.indiatimes.com/life-style/health-fitness/health-news/suffering-from-endometriosis-or-adenomyosis-heres-the-difference/photostory/98910929.cms	1
https://drauziovarella.uol.com.br/mulher/adenomiose-causas-sintomas-e-possiveis-tratamentos/	1	https://www.milann.co.in/blogs/adenomyosis	1
https://drbrunobonilha.com.br/procedimento-adenomiose/	1	https://www.glamourmagazine.co.uk/article/difference-between-endometriosis-and-adenomyosis	1
https://www.terra.com.br/vida-e-estilo/saude/uma-em-cada-10-mulheres-tem-adenomiose-conheca-endometriose-do-utero,bfd14c8558580242c5ea503a7 9f7eaed0ma8g25v.html	1	https://ferticity.com/endometriosis-adenomyosis-treatment-in-delhi	1
https://clinicabuzzini.com.br/ja-ouviu-falar-em-adenomiose/	1	https://carnegiewomenshealth.com/blog/the-difference-between-adenomyosis-and-endometriosis/	1
https://drathaisvazquez.com.br/2023/02/13/adenomiose-o-que-e-isso/	1	https://www.newlife-ivf.co.uk/blog/what-is-adenomyosis	1
href="https://artmedicina.com.br/utero-com-adenomiose/">https://artmedicina.com.br/utero-com-adenomiose/">href="https://artmedicina.com.br/utero-com-adenomiose/	1	https://www.fertilitytexas.com/endometriosis-versus-adenomyosis/	1
https://sogimig.org.br/adenomiose/conteudo-para-pacientes/	1	https://fertiltree.com/blogs/bulky-uterus/	1
https://feitoparaela.com.br/2023/04/25/adenomiose-voce-sabe-o-que-e-esta-doenca-que-pode-causar-infertilidade/	1	https://timesofindia.indiatimes.com/blogs/voices/adenomyosis-causes-and-treatment/	1
https://drapriscilamatsuoka.com.br/a-adenomiose-pode-virar-cancer-uterino/	3	https://www.chandigarhayurvedcentre.com/blog/adenomyosis-ayurvedic-treatment/	1
https://oscarduarte.com.br/adenomiose.html	3	https://womensclinicbtm.com/adenomyosis/	1
https://unih.clinic/adenomiose-causas-sintomas-e-tratamentos/	3	https://www.centerofendometriosis.com/blog/endometriosis-vs-adenomyosis-understanding-the-difference/	1
https://www.tuasaude.com/adenomiose/	3	https://www.lotusmedics.com.au/blog/adenomyosis-vs-fibroids/	1
https://www.mdsaude.com/ginecologia/menstruacao/adenomiose/	3	https://www.prepladder.com/neet-pg-study-material/obstetrics-and-gynaecology/endometriosis-and-adenomyosis-neet-pg	1
https://adrianadegoes.med.br/adenomiose/	3	https://www.brahmhomeo.com/disease-details/adenomyosis-treatment-in-homeopathy/161	1
https://ipgo.com.br/adenomiose-completo/#:~:text=8%3A27%20pm-,O%20que%20 %C3%A9%20a%20Adenomiose%3F,da%20Zona%20 Juncional%20(ZJ).	5	https://www.verywellhealth.com/adenomyosis-vs-endometriosis-5248776	3
		https://www.shreeivfclinic.com/blogs/what-is-the-meaning-of-adenomyosis/	3
		https://www.indiraivf.com/infertility-problems/adenomyosis	3
		https://www.msdmanuals.com/home/women-s-health-issues/miscellaneous-gynecologic-abnormalities/uterine-adenomyosis	3
		https://www.theendometriosisfoundation.org/adenomyosis	3
		https://www.drharris.com.au/adenomyosis-obstetrician-gynaecologist-perth/	3
		https://www.cosmopolitan.com/uk/body/health/a40252558/adenomyosis-vs-endometriosis/	3
		https://ayu.health/blog/distinguishing-endometriosis-and-adenomyosis-understanding-differences/	3
		https://insixteenyears.com/adenomyosis/	3
		https://www.nhsinform.scot/healthy-living/womens-health/girls-and-young-women-puberty-to-around-25/periods-and-menstrual-health/adenomyosis/	3
		https://my.clevelandclinic.org/health/diseases/14167-adenomyosis	5
		https://www.kegel8.co.uk/help-and-advice/adenomyosis	5
		https://www.epworth.org.au/our-services/endometriosis-centre/related/adenomyosis	5
		https://momotaroapotheca.com/blogs/vaginal-wellness/chronic-pelvic-pain-adenomyosis-symptoms-treatment-diagnosis	5

Of the 32 sites, only 10 were considered accurate (31.25%), while the other 22 were not accurate. Among the sites considered accurate (score of 8 or higher), 1 site received a score of 11 (out of 12), 3 sites scored 10, 4 sites scored 9, and 2 sites scored 8. Among the sites considered not accurate, 8 sites received a score of 7, 4 sites scored 6, 9 sites scored 5, and 1 site scored 3. The average accuracy score of the sites in English was 6.9, which was higher than the average of the sites in Portuguese. Of the 32 sites, only 2 were considered credible, both scoring 9 (out of 10). The remaining 30 sites were considered not credible: 5 sites scored 6, 3 sites scored 5, 9 sites scored 4, 6 sites scored 3, 4 sites scored 2, 2 sites scored 1, and 1 site scored 0. The average credibility score of the sites in English was 3.968, which was lower than the average of the sites in Portuguese.

All English-language websites included a definition of endometriosis. The risk factors were described on 29 websites. Regarding symptoms, all websites provided a description, and diagnosis was addressed on 26 websites. Medical treatment was described on 28 websites, while surgical treatment was mentioned on 25.

A total of 451 sites were analyzed, considering the sites in English and Portuguese, with 51 included and 400 excluded, mainly due to social media. The average credibility score was 4.196, with 47 sites lacking credibility (92.15%). All included sites were rated as very difficult or difficult to read, requiring higher education (22 sites required ongoing college, 29 sites required a college graduate). Only 5 sites were deemed of good quality according to DISCERN. Of these, 30 sites received a score of 1, 16 sites received a score of 3, and 5 sites received a score of 5. The average accuracy was 6.803, with 16 sites considered accurate and 35 not.

## DISCUSSION

The search for online information is often a first step for patients seeking clarification of their symptoms, particularly in resource-limited places or where access to specialist care is delayed. When the available information is inaccurate or lacking in scientific rigor, it not only delays diagnosis and treatment but may also foster anxiety, lead to harmful self-management practices, and impair trust in healthcare professionals^4,6^.

In the Portuguese websites, the main reason for exclusion was social media content, suggesting a possible prevalence of social media as a primary platform for discussing medical conditions. In contrast, most of the excluded English websites were categorized as medical literature, which may hinder comprehension and even lead to readers abandoning the content altogether.

This study evaluated the quality, credibility, and accuracy of online lay literature on adenomyosis, revealing an alarming prevalence of low-quality information. Out of 451 analyzed websites, only 5 (1.1%) were rated as high-quality according to the DISCERN instrument, while the vast majority (92.1%) lacked credibility. These findings align with prior research on other gynecological conditions, which consistently highlights the widespread presence of misleading or incomplete health content on the internet^5,7^.

The results of this study are consistent with the existing literature. A meta-analysis of 153 studies evaluating the quality of 11,785 health-related websites aimed at the general public found that none of the sources were rated as “excellent” according to the DISCERN criteria. Between 37 and 79% were considered to be of good quality, while the remainder were classified as poor^13^. In that study, websites were categorized by medical specialty, and in addition to DISCERN, the HONcode—a code of ethics developed to guide site managers in providing high-quality, objective, and transparent medical information online—was also used to assess website quality. Among the evaluated websites, those related to oncology and internal medicine had the highest rate of HONcode certification (30%), whereas gynecology and obstetrics had the lowest (11%).

This is particularly concerning, as it suggests that conditions such as adenomyosis, along with other gynecological disorders, are being inadequately addressed on the internet, potentially undermining women’s healthcare. Another cross-sectional literature review of published studies on online health information, which examined trends in the use of health-related websites, concluded that the individuals most likely to seek health information online were women, younger people, students, and working professionals^14^. This highlights how women, in particular, may be especially vulnerable in this context. The quality of online health information requires significant improvement—an issue that should be prioritized by policymakers as well as public and private institutions.

Moreover, the readability analysis indicated that most texts required a high level of formal education, further limiting accessibility. A systematic review investigating differences in how people with varying levels of health literacy evaluate online information found that low health literacy, education level, and related skills are negatively associated with both the ability to critically assess and the trust placed in online health information^15^. These barriers affect mainly vulnerable populations and contradict recommendations from international institutions advocating for inclusive, patient-centered communication in women’s health^16^.

## CONCLUSION

The majority of sites found in English and Portuguese on Google are unreliable sources regarding adenomyosis, often classified as “low quality,” primarily failing to address treatment comprehensively.

## Data Availability

The datasets generated and/or analyzed during the current study are available from the corresponding author upon reasonable request.
